# Photosynthesis under Biotic and Abiotic Environmental Stress

**DOI:** 10.3390/cells11243953

**Published:** 2022-12-07

**Authors:** Marian Brestic, Suleyman I. Allakhverdiev

**Affiliations:** 1 Institute of Plant and Environmental Sciences, Slovak University of Agriculture, A. Hlinku 2, 94976 Nitra, Slovakia.; 2 K.A. Timiryazev Institute of Plant Physiology, Russian Academy of Sciences, Botanicheskaya Street 35, 127276 Moscow, Russia

Photosynthesis is a unique process that has shaped life on our planet and created the conditions for all known life forms. During evolution, plant species and photosynthetic forms have emerged, and partial mechanisms have been optimized to work within a range of environmental conditions. However, variable environmental conditions caused by climate change, environmental pollution, and biotic factors significantly limit growth, biomass production, and plant reproduction.

By improving our understanding of the reactions and partial processes of photosynthesis in a changing or stressful environment, we can predict how plants will function in different climate-change scenarios when exposed to abiotic and biotic stressors, as well as how to improve their adaptability.

In this Special Issue of *Cells*, we present a collection of articles dedicated to various aspects of photosynthesis, representing research into the effects of abiotic and biotic stresses that can adversely affect structures and function, metabolism, and photochemical and biochemical processes from the molecular to the whole-plant level.

Cyanobacteria are prokaryotic photosynthetic organisms with considerable agrobiotechnological potential. They are used to produce biofertilizers, and contribute significantly to plant drought resistance and nitrogen enrichment in soil [1]. Sadvakasova et al. sought, isolated, and investigated nitrogen-fixing cyanobacterial strains in rice fields and evaluated the effect of Mo and Fe on photosynthetic and nitrogenase activities under nitrogen starvation. Cyanobacterial isolates isolated from rice paddies in Kazakhstan were identified as *Trichormus variabilis K-31 (MZ079356), Cylindrospermum badium J-8 (MZ079357), Nostoc sp. J-14 (MZ079360), Oscillatoria brevis SH-12 (MZ090011),* and *Tolypothrix tenuis J-1 (MZ079361).* The study of the influence of various concentrations of Mo and Fe on photosynthetic and nitrogenase activities under conditions of nitrogen starvation revealed the optimal concentrations of metals that stimulate the studied parameters [1].

The quality of light is an important factor in regulating plant growth and development during ontogenesis, including germination, photomorphogenesis, flowering induction, etc. Pashkovskiy et al. studied the influence of light of different qualities on the growth, gas exchange, fluorescence indices of Chl a, and expression of key light-dependent genes of Pinus sylvestris L. seedlings. In plants growing under red light (RL), the biomass of needles and root systems increased by more than two and three times, respectively, compared with those of the white fluorescent light (WFL) control. Meanwhile, the rates of photosynthesis and respiration in RL and blue light (BL) plants were lower than those of blue–red-light (BRL) plants, and the difference between the rates of photosynthesis and respiration, which characterizes the carbon balance, reached its maximum under RL [2].

According to the authors, RL influenced the number of xylem cells, activated the expression of genes involved in the transduction of cytokinin and auxin signals, and reduced the expression of the gene encoding the transcription factor phytochrome-interacting factor 3 (PIF3). It was suggested that RL-induced activation of key genes of cytokinin and auxin signaling might indicate a phytochrome-dependent change in cytokinin and auxin activity [2].

The effects of different nanoparticles and their combined effects on terrestrial plants have not been thoroughly investigated. This subject is a challenge for modern ecotoxicology. Cerium oxide nanoparticles (CeO2 NPs) and zinc oxide nanoparticles (ZnO NPs) are emerging pollutants [3]. In the study by Skiba et al., Pisum sativum L. plants were exposed to either CeO2 NPs or ZnO NPs alone or mixtures of these nano-oxides (at two concentrations: 100 and 200 mg/L) [3]. The authors concluded that CeO2 NPs moderate ZnO NP toxicity by protecting the photosynthetic apparatus in Pisum sativum leaves from oxidative stress triggered by Zn. Additionally, they observed that both nano-oxides affected nutrient uptake and transport at all concentrations applied. These results indicate that the free-radical scavenging properties of CeO2 NPs mitigate the toxicity induced by ZnO NPs [3].

Light plays an essential role in photosynthesis; however, its excess can cause damage to cellular components. Photosynthetic organisms thus developed a set of photoprotective mechanisms (e.g., nonphotochemical quenching, photoinhibition) that can be studied by classic biochemical and biophysical methods in cell suspensions. Here, we combined these bulk methods with single-cell identification of microdomains in the thylakoid membrane during high light (HL)-stress [4]. Canonico et al. used Synechocystis sp. PCC 6803 cells with yellow fluorescent protein (YFP)-tagged photosystem I and identified a multiphase response of cyanobacteria to HL stress with three main phases: fast, intermediate, and slow [4]. The authors observed the accumulation of myxoxanthophyll and more even spatial distribution of photosystems and phycobilisomes between microdomains. They suggest that the overall carotenoid increase during HL stress could be involved either in direct photoprotection (e.g., in ROS scavenging) and/or could play an additional role in maintaining the optimal distribution of photosystems in the thylakoid membrane to attain efficient photoprotection [4].

PSI photoinhibition is usually avoided through P700 oxidation. Without this protective mechanism, excess light represents a potentially lethal threat to plants. PGR5 is suggested to be a major component of cyclic electron transport around PSI and is important for P700 oxidation in angiosperms. The Arabidopsis PGR5-deficient mutant pgr5-1 is incapable of P700 oxidation regulation and has been used in numerous photosynthetic studies. However, Wada et al. revealed that pgr5-1 was a double mutant with exaggerated PSI photoinhibition [5]. pgr5-1 significantly reduced growth compared to the newly isolated PGR5-deficient mutant pgr5hope1. The introduction of PGR5 into pgr5-1 restored P700 oxidation regulation but maintained a pale-green phenotype, indicating that pgr5-1 had additional mutations. Both pgr5-1 and pgr5hope1 tended to cause PSI photoinhibition by excess light, but pgr5-1 exhibited an enhanced reduction in PSI activity [5].

In the work by Fatima et al., the combined response of exclusion of solar ultraviolet radiation (UV−A+B and UV−B) and static magnetic field (SMF) pretreatment was studied on soybean (Glycine max) leaves using synchrotron imaging [6]. The solar UV exclusion results suggested that ambient UV caused a reduction in leaf growth, which ultimately reduced photosynthesis in soybean seedlings, while SMF treatment enhanced leaf growth along with photosynthesis, even in the presence of ambient UV-B stress. The results suggested that SMF pretreatment of seeds diminishes the ambient UV-induced adverse effects on soybean [6].

Principal component analysis of foliar pigment composition revealed that Malva was similar to fast-growing annuals, while Lemna was similar to slow-growing evergreens [7]. Overall, Lemna exhibited traits reminiscent of those of its close relatives in the family Araceae, with a remarkable ability to acclimate to both deep shade and full sunlight. Overall, duckweed exhibits a combination of traits of fast-growing annuals and slow-growing evergreens with foliar pigment features that represent an exaggerated version of that of terrestrial perennials combined with an unusually high growth rate [7]. Duckweed’s ability to thrive under a wide range of light intensities can support success in a dynamic light environment with periodic cycles of rapid expansion [7].

The efficacy of microbial endophytes in promoting plant growth and their comparison with exogenously applied hormones have not been investigated until now. The aim of the work by Ismail et al. was the isolation, identification, and characterization of bacterial and fungal endophytes from the roots of the *Phaseolus vulgaris* plant and the exploration of their potentiality compared to two common exogenously applied hormones on the growth and biochemical properties of *P. vulgaris* plants to explore the possibility of applying these microbial isolates as biofertilizers for the improvement of the growth performance and metabolites of crops. Their results indicated that the endophyte *Brevibacillus agri* (PB5) provides high potential as a stimulator for the growth and productivity of common bean plants [8].

Photosynthesis is an important target of action of numerous environmental factors; in particular, stressors can strongly affect photosynthetic light reactions. Considering the reactions of photosynthetic light to electron and proton transport, it can be supposed that an extremely low-frequency magnetic field (ELFMF) may influence these reactions; however, this problem has been weakly investigated [9]. Sukhov et al. experimentally tested a hypothesis about the potential influence of ELFMF on photosynthetic light reactions in wheat and pea seedlings [9]. The authors showed that ELFMF with Schumann resonance frequencies could influence photosynthetic light processes; however, this effect depends on the plant species (wheat or pea) and the type of treatment (short-term or chronic).

The effect of abiotic factors on the abundance and photosynthetic performance of airborne Cyanobacteria and microalgae isolated from the southern Baltic Sea region was studied by Wisniewska et al. [10]. Their experiments suggest that the adaptive abilities of microorganisms—particularly those producing toxins—may contribute to the spread, potentially increasing human exposure to their negative health effects. Any distinctive adaptations of the genera give them an additional competitive advantage and a greater chance for territorial expansion [10].

Black spot disease, caused by *Alternaria brassicicola* in *Brassica* species, is one of the most devastating diseases worldwide, especially since there is no known fully resistant *Brassica cultivar*. In this context, Macioszek et al. provides a report on the susceptible interaction between *B. oleracea var. capitata f. alba* (cultivar ‘*Glory of Enkhuizen*’) and *A. brassicicola*, both from the fungus and host plant perspectives [11]. The authors focused on the details of fungal development and colony formation and plant-cell reactions during infection at both light and transmission microscopy levels. Ultrastructural, molecular, physiological, and transcriptional analyses of infected leaves revealed photosynthesis as the most downregulated process from the onset of the infection [11]. This finding should be taken into consideration in further research when composing a strategy for the management of black spot disease.

One of our most striking observations concerns the significant difference between the physiological responses of different Synechococcus sp. phenotypes to changeable environmental conditions. The main aim of Śliwińska-Wilczewska’s paper was to determine the acclimatization capacity of three Baltic phenotypes of *Synechococcus sp*. [12], an important link in forecasting future changes in the occurrence of these organisms in the context of global warming. Furthermore, the study focused on the effect of irradiance, temperature, and their mutual interactions on the content and proportions of cell-specific photosynthetic pigments of the examined cyanobacterial phenotypes. The authors reported that the detailed characterization of the quantitative and qualitative composition of pigments is important to determine the level of acclimatization of the examined phenotypes of cyanobacteria to specific environmental conditions. Awareness of the biology and physiology of these organisms obtained by capturing their reactions to various environmental factors is important for forecasting their possible expansion [12].

A review by Allakhverdiev et al. provides a comprehensive overview of Raman spectroscopy (RS) and its modifications applied to biological and medical research [13]. In this review, several existing studies on biological, medical, analytical, photosynthetic, and algal research using RS have been analyzed and presented. RS is used for a variety of studies in animals and human research. A greater focus on the application of RS in algal research will be beneficial for biotechnological purposes and general knowledge of the mechanisms of biomolecule interactions in algae under natural/environmental conditions. RS is a very attractive, advantageous, and promising approach for algal research [13].

Plant seeds are an essential input in agriculture; however, during their developmental stages, seeds can be negatively affected by environmental stresses, which can adversely affect seed vigor, seedling establishment, and crop production. Seeds resistant to high salinity, droughts, and climate change can result in higher crop yield [14]. The major findings suggested in this review by Shelar et al. refer to nano-priming as an emerging seed technology for sustainable food amid growing demand from the increasing global population [14]. This novel technology could influence the crop yield and ensure the quality and safety of seeds in a sustainable way. When nano-primed seeds are germinated, they undergo a series of synergistic events due to their enhanced metabolism: modulating biochemical signaling pathways, triggering hormone secretion, and reducing reactive oxygen species, leading to improved disease resistance. In addition to providing an overview of the challenges and limitations of seed nanopriming technology, this review also describes some of the emerging nanoseed priming methods for sustainable agriculture and other technological developments using cold plasma technology and machine learning [14].

Abiotic stresses, such as drought, salinity, heavy metals, variations in temperature, and ultraviolet (UV) radiation, are antagonistic to plant growth and development, resulting in an overall decrease in plant yield [15]. These stresses have direct effects on the rhizosphere, severely affecting root growth and thereby affecting plant growth, health, and productivity. However, the growth-promoting rhizobacteria that colonize the rhizosphere/endorhizosphere protect the roots from the adverse effects of abiotic stress and facilitate plant growth by various direct and indirect mechanisms [15]. In the rhizosphere, plants constantly interact with thousands of these microorganisms, yet it is unclear when and how these complex root, rhizosphere, and rhizobacteria interactions occur under abiotic stresses. A review by Khan et al. focuses on root–rhizosphere and rhizobacterial interactions during stress, how roots respond to these interactions, and the role of rhizobacteria under these stresses. Furthermore, the review focuses on the underlying mechanisms employed by rhizobacteria to improve root architecture and plant tolerance to abiotic stresses [15].

The original research and review articles collected in this Special Issue will be of interest to a broad audience of scientists studying photosynthesis under conditions of biotic and abiotic environmental stress, addressing all aspects of photosynthesis, including regulatory mechanisms, as well as their value in agriculture, forestry, ecology, and biotechnology. We hope that interdisciplinary applications of knowledge in this Special Issue will stimulate future research.

We would like to express our sincerest thanks to our readers, authors, anonymous peer reviewers, editors, and all the people working for the journal, all of whom have made substantial contributions to this Special Issue. This would not have been possible without your support. For the details of the Special Issue “Photosynthesis under Biotic and Abiotic Environmental Stress “please click here (https://www.mdpi.com/journal/cells/special_issues/Photosynthesis_Biotic_Abiotic_Environmental_Stress, accessed on 1 June 2020).

## Data Availability

The datasets generated during and/or analyzed during the current study are available from the corresponding author on reasonable request.

## References

[1] Sadvakasova A.K., Kossalbayev B.D., Token A.I., Bauenova M.O., Wang J., Zayadan B.K., Balouch H., Alwasel S., Leong Y.K., Chang J.-S. (2022). Influence of Mo and Fe on Photosynthetic and Nitrogenase Activities of Nitrogen-Fixing Cyanobacteria under Nitrogen Starvation. Cells.

[2] Pashkovskiy P., Kreslavski V.D., Ivanov Y., Ivanova A., Kartashov A., Shmarev A., Strokina V., Kuznetsov V.V., Allakhverdiev S.I. (2021). Influence of Light of Different Spectral Compositions on the Growth, Photosynthesis, and Expression of Light-Dependent Genes of Scots Pine Seedlings. Cells.

[3] Skiba E., Pietrzak M., Glińska S., Wolf W.M. (2021). The Combined Effect of ZnO and CeO2 Nanoparticles on Pisum sativum L.: A Photosynthesis and Nutrients Uptake Study. Cells.

[4] Canonico M., Konert G., Crepin A., Šedivá B., Kaňa R. (2021). Gradual Response of Cyanobacterial Thylakoids to Acute High-Light Stress—Importance of Carotenoid Accumulation. Cells.

[5] Wada S., Amako K., Miyake C. (2021). Identification of a Novel Mutation Exacerbated the PSI Photoinhibition in pgr5/pgrl1 Mutants; Caution for Overestimation of the Phenotypes in Arabidopsis pgr5-1 Mutant. Cells.

[6] Fatima A., Kataria S., Agrawal A.K., Singh B., Kashyap Y., Jain M., Brestic M., Allakhverdiev S.I., Rastogi A. (2021). Use of Synchrotron Phase-Sensitive Imaging for the Investigation of Magnetopriming and Solar UV-Exclusion Impact on Soybean (Glycine max) Leaves. Cells.

[7] Stewart J.J., Adams W.W., López-Pozo M., Doherty Garcia N., McNamara M., Escobar C.M., Demmig-Adams B. (2021). Features of the Duckweed Lemna That Support Rapid Growth under Extremes of Light Intensity. Cells.

[8] Ismail M.A., Amin M.A., Eid A.M., Hassan S.E.-D., Mahgoub H.A.M., Lashin I., Abdelwahab A.T., Azab E., Gobouri A.A., Elkelish A. (2021). Comparative Study between Exogenously Applied Plant Growth Hormones versus Metabolites of Microbial Endophytes as Plant Growth-Promoting for Phaseolus vulgaris L.. Cells.

[9] Sukhov V., Sukhova E., Sinitsyna Y., Gromova E., Mshenskaya N., Ryabkova A., Ilin N., Vodeneev V., Mareev E., Price C. (2021). Influence of Magnetic Field with Schumann Resonance Frequencies on Photosynthetic Light Reactions in Wheat and Pea. Cells.

[10] Wiśniewska K., Śliwińska-Wilczewska S., Lewandowska A., Konik M. (2021). The Effect of Abiotic Factors on Abundance and Photosynthetic Performance of Airborne Cyanobacteria and Microalgae Isolated from the Southern Baltic Sea Region. Cells.

[11] Macioszek V.K., Gapińska M., Zmienko A., Sobczak M., Skoczowski A., Oliwa J., Kononowicz A.K. (2020). Complexity of Brassica oleracea–Alternaria brassicicola Susceptible Interaction Reveals Downregulation of Photosynthesis at Ultrastructural, Transcriptional, and Physiological Levels. Cells.

[12] Śliwińska-Wilczewska S., Konarzewska Z., Wiśniewska K., Konik M. (2020). Photosynthetic Pigments Changes of Three Phenotypes of *Picocyanobacteria Synechococcus* sp. under Different Light and Temperature Conditions. Cells.

[13] Allakhverdiev E.S., Khabatova V.V., Kossalbayev B.D., Zadneprovskaya E.V., Rodnenkov O.V., Martynyuk T.V., Maksimov G.V., Alwasel S., Tomo T., Allakhverdiev S.I. (2022). Raman Spectroscopy and Its Modifications Applied to Biological and Medical Research. Cells.

[14] Shelar A., Singh A.V., Maharjan R.S., Laux P., Luch A., Gemmati D., Tisato V., Singh S.P., Santilli M.F., Shelar A. (2021). Sustainable Agriculture through Multidisciplinary Seed Nanopriming: Prospects of Opportunities and Challenges. Cells.

[15] Khan N., Ali S., Shahid M.A., Mustafa A., Sayyed R.Z., Curá J.A. (2021). Insights into the Interactions among Roots, Rhizosphere, and Rhizobacteria for Improving Plant Growth and Tolerance to Abiotic Stresses: A Review. Cells.

